# Use of Apomorphia in Cases of Poisoning

**Published:** 1883-12-20

**Authors:** Amand Routh

**Affiliations:** M.D., B. S., M. R. C. P., London


### USE OF APOMORPHIA IN CASES OF POISONING.

By AMAND ROUTH, M.D., B.S., M.R.C.P., LOND.

Those liable to be called to cases of poisoning are always glad to haye an agent handy which, not in itself lowering, will produce prompt emesis, especially in those cases where the jaws are rigidly clenched and the stomach pump absent or inadmissible. This agent,

I am sure, we have in apomorphia, an alkaloid which Dr. W. Murrell has brought before the profession. Though a derivative of morphia, it has no norcotic effects in the doses required to cause emesis. Dr. Murrell recommends it to be kept in a solution of 1 in 50 strength, and to be given subcutaneously in doses of from 3½ to 10 minims (1-15 to 1-5 grain.) Emesis occurs in from two to five minutes, the contents of the stomach being usually voided in one rush without previous nausea, but with violent and visible muscular action of the stomach walls. The following two cases will serve to show its utility.

CASE 1.—I was sent for to see Mrs. S——, who was said to have swallowed a white powder and to be then dead. I found her on the floor, doubled up, jaws and hands clenched, blood and froth at mouth, respiration seemed absent and pulse barely perceptible. She had not vomited. Though evidently dying, I injected five minims of the above solution into her arm, keeping my hand on the pulse. It two minutes and a half by the watch the stomach evacuated its contents with a rush, whilst the pulse seemed to rally for an instant and then finally ceased. Oxalic acid was proved to have been the poisou used, and at the post-mortem about two drachms only of fluid were found in the stomach.

CASE 2.—A lady, a dipsomaniac, had obtained access to the wine cellar, and had swallowed straight off two bottles and a half of brandy. She then put the corks in her pocket, hid the bottles, put on her clothes and went out for a walk with her footman. She walked quite steadily for three hundred yards, when she dropped down insensible and was carried home in a cab. On arrival, ten minutes after, I found her comatose, not able to be roused, respiration stertorous and infrequent, pupils dilated and insensible, jaws clenched, pulse slow and intermitting, two or three beats in every eight. Her stomach was full of fluid. I injected 3½ drops of the solution, and in exactly three minutes and a half about a pint of alcoholic liquor was expelled, and altogether in about five minutes a quart (measured) of hardly altered brandy was vomited. The pulse and respiration now improved, the pupils becoming slightly sensible, and I left her for two hours, by which time she could be roused temporarily. After twelue hours' sleep she awoke none the worse.

Apomorphia fails to cause emesis during chloroform narcotism, but no other drug seems to be antagonistic to it, and there is no reason why it should not be used to get rid of even morphia itself. In the dyspnœa of chronic bronchitis, emesis from apomorphia produces temporary relief. If only the certainty, rapidity and absolute safety of apomorphia were known, it would undoubtedly form part of every practitioner's paraphernalia.

[Dr. Routh has since (Lancet, Dec. 30) received a note from Dr. Murrell stating that it was Dr. Gee who first introduced the use of morphia to the profession.]—*Lancet, Dec. 23, 1882, p. 1073.*

